# The Role of Autophagy and Apoptosis in the Combined Action of Plasma-Treated Saline, Doxorubicin, and Medroxyprogesterone Acetate on K562 Myeloid Leukaemia Cells

**DOI:** 10.3390/ijms24065100

**Published:** 2023-03-07

**Authors:** Tatyana Pavlik, Victoria Gudkova, Darya Razvolyaeva, Marina Pavlova, Nadejda Kostukova, Lilia Miloykovich, Leonid Kolik, Evgeny Konchekov, Nikolay Shimanovskii

**Affiliations:** 1 Prokhorov General Physics Institute of the Russian Academy of Sciences, 119991 Moscow, Russia; 2 Faculty of Biomedicine, Pirogov Russian National Research Medical University, 117997 Moscow, Russia; 3 Faculty of Science, Peoples Friendship University of Russia (RUDN University), 117198 Moscow, Russia

**Keywords:** plasma-treated solutions (PTS), medroxyprogesterone acetate (MPA), doxorubicin, autophagy, apoptosis

## Abstract

The anti-cancer properties of plasma-treated solutions (PTS) and their interaction with drugs are one of the most popular topics in modern plasma medicine. Our research involved comparing the effects of four physiological saline solutions (0.9% NaCl, Ringer’s solution, Hank’s Balanced Salt Solution, Hank’s Balanced Salt Solution with amino acids added in concentrations observed in the human blood) treated with cold atmospheric plasma and studying the combined cytotoxic effect of PTS with doxorubicin and medroxyprogesterone acetate (MPA). Analysis of the effect of the studied agents on the formation of radicals in the incubation medium, the vitality of K562 myeloid leukaemia cells, and the processes of autophagy and apoptosis in them revealed two key findings. The first is that when using PTS and doxorubicin-containing PTS, autophagy is the predominant process in cancer cells. The second is that combining PTS with MPA enhances apoptotic processes. It was hypothesised that while autophagy is stimulated by the accumulation of reactive oxygen species in the cell, apoptosis is stimulated through specific cell progesterone receptors.

## 1. Introduction

Cold plasma has already proved its effectiveness in the destruction of cancer cells in vitro [1,2,3,4] and in vivo [2,4]. In parallel with the direct effect of cold plasma on biological objects, the properties of solutions treated with such plasma and their possible impact on biological objects are currently being investigated by various authors [5,6,7,8]. The use of such plasma-treated solutions in oncology is of the greatest interest since, in most cases, cancer cells are not readily accessible for direct cold plasma treatment without surgical interventions.

Currently, changes in the composition of various aqueous plasma-treated solutions (PTS) have been analysed, including distilled and deionised water [9,10,11,12], phosphate-buffered saline [12,13,14], sodium chloride solution [10,12,13,14], electrolyte solution [13,15], Ringer’s lactate solution [13,14,15,16], glucose solution [13], hydroxyethyl starch solution [13,14], and gelafundin solution [13]. A change in the structure of some amino acids in solution under the action of cold plasma has been observed [17,18]. Cold plasma treatment by Ringer’s lactate solution produces pyruvate, acetate, glyoxylate, and 2,3-dimethyl tartrate, with the latter playing an active role in the selective cytotoxic effect on glioblastoma cells [19].

Isotonic solutions that can be injected directly into the blood have aroused the greatest interest in terms of their potential use in anti-cancer treatment. The anti-cancer activity of the above solutions was studied on cell cultures and showed different cytotoxic properties [13,15,16]. Most of the studies are devoted to plasma treatment of the nutrient medium in which the cells are placed [20,21,22] or the nutrient medium in which the cells are already located [21,23,24,25]. Although the nutrient medium has a complex composition and the most effective cytotoxic effect, it is not prescribed for medical use. This article is therefore devoted to discussing and analysing the changes that occur under the influence of cold plasma in intravascular-administered physiological saline solutions.

Various literature has shown how peroxynitrite (ONOO^−^) plays an important role in the biological action of cold plasma [26,27,28]. With a decrease in the pH of a solution treated with cold plasma from 7.4 to 6.0, its interaction with a tyrosine solution significantly reduces the formation of nitrotyrosine, a product of the interaction of tyrosine with ONOO^−^ derivatives [27]. ONOO^−^ in a solution treated with cold plasma is much better preserved at alkaline pH than at neutral or acidic pH [26].

Solutions treated with cold plasma acquired cytotoxic properties that became more pronounced with increasing treatment time [14,16,29]. The formation of proteins involved in programmed cell death and DNA damage under the action of PTS have been well documented [30]. Moreover, the selective cytotoxic effect of PTS on specific cancer cell lines has been demonstrated in various works [20]. There have also been studies on the synergistic effect of a combination of PTS and cytostatic drugs, with the best synergy found in the case of a combination with cisplatin [21,29].

The use of plasma-treated solutions in combination with various medications is currently one of the most important areas of research in plasma medicine. However, the effects of combinations with anti-cancer drugs are still very poorly studied. No comparison has been made, for example, between physiological saline solutions and solutions of organic substances treated with cold plasma.

Doxorubicin is a well-known cytostatic drug used in the clinic for the treatment of oncological diseases. Its mechanism of action is based on damage to the DNA of tumour cells and triggering the generation of reactive oxygen and nitrogen species (RONS) [31,32]. Unfortunately, like any cytostatic, doxorubicin has side effects. The most global of them is the cardiotoxic effect [31]. The use of doxorubicin in combination with anti-cancer drugs with a different mechanism of action would reduce the therapeutic dose of doxorubicin, and thus reduce its side effects.

Progestins have also shown anti-tumour effects on cells having receptors for progesterone or glucocorticoids [33,34,35]. Medroxyprogesterone acetate (MPA) have been successfully used to treat advanced breast cancer, endometrial cancer, prostate cancer, and endometrial hyperplasia [36]. Since the cytotoxic effect of MPA on tumour cells occur through interaction with specific receptors, its use for the treatment of oncological diseases looks attractive. Using drugs acting only on certain morphological groups of cells, it is possible to avoid attacking healthy dividing cells, for example, bone marrow.

Both doxorubicin and MPA showed selective independent effects on cancer cells [37,38]. One of our tasks was to investigate the possibility of enhancing the cytotoxic effect of anti-tumour drugs using PTS as an example of doxorubicin and MPA.

Additionally, in this work, a comparison was made between the chemical changes and the cytotoxic effect of solutions similar in composition to human blood plasma that has been pre-treated with cold plasma. For this, 0.9% NaCl solution, Ringer’s solution containing only inorganic salts, Hank’s Balanced Salt Solution (HBSS) containing inorganic salts and glucose, and HBSS with the addition of amino acids at concentrations that are normally observed in human blood were selected.

## 2. Results and Discussion

The foremost procedure in the experiment was to evaluate the change in the composition of the studied solutions when subjected to cold plasma to be able to interpret the mechanism of its effect on cultured cells.

### 2.1. Changes in the Chemical Characteristics of Solutions after Cold Plasma Treatment

**Figure 1:**
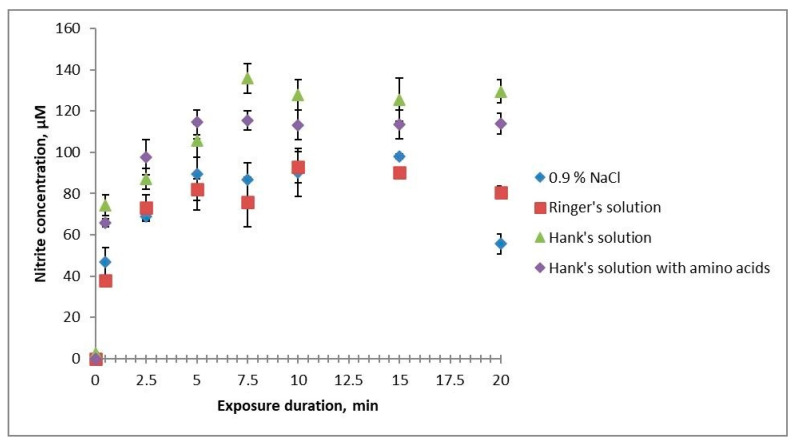
Dependence of the concentration of nitrite ions in the studied solutions on their cold plasma treatment times.

Treating the studied solutions with cold plasma generated nitrite ions in them (Figure 1). A discrepancy in the results in terms of the effective action times for different solutions was observed after 5 min of treatment: HBSS and HBSS with amino acids accumulated more nitrites, and their concentrations remained stable with increasing treatment time; 0.9% NaCl solution and Ringer’s solution had a 30% less nitrite concentration that began decreasing after 15 min of treatment. At that, the concentration of nitrites in Ringer’s solution decreased more slowly than in 0.9% NaCl solution. This difference may be due to the buffer properties of the solutions, as where they are weak, nitrites are oxidised to nitrates.

**Figure 2:**
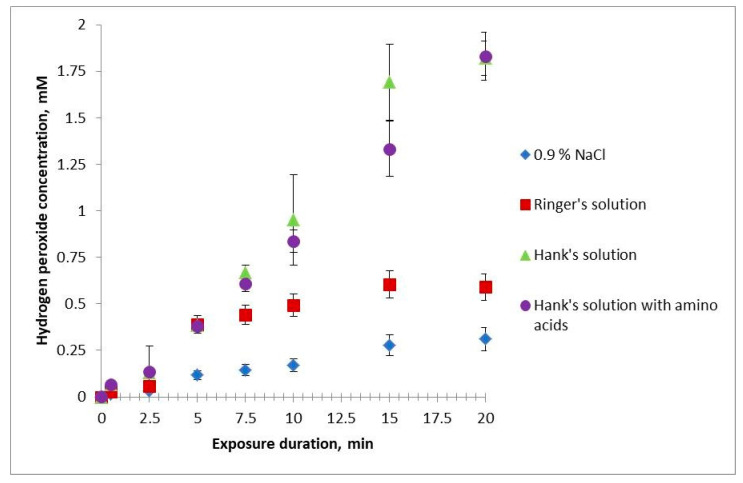
Dependence of the concentration of hydrogen peroxide in the studied solutions on the times of their treatment with cold plasma.

The concentration of hydrogen peroxide in HBSS and HBSS with amino acids increases linearly, directly proportional to the treatment time, and is the same for both solutions at all treatment times (Figure 2). The effective action time of hydrogen peroxide in NaCl solution and Ringer’s solution is similar to that in HBSS and HBSS with amino acids at a treatment time of 2.5 min or less. At 5 min of treatment, the concentration of hydrogen peroxide in the NaCl solution is significantly less than in other solutions. At 7.5 min of treatment, the curves for HBSS and Ringer’s solution diverge. In the treatment time range of 10–20 min, the concentration of hydrogen peroxide in the NaCl solution and Ringer’s solution is significantly less than in HBSS and HBSS with amino acids. The concentration of hydrogen peroxide in the NaCl solution and Ringer’s solution first increases linearly, then begins to level out after 7.5 min of treatment. At that, it remains higher in Ringer’s solution than in the NaCl solution.

Hypochlorite was not detected in all the studied solutions at all times of treatment with cold plasma.

**Figure 3:**
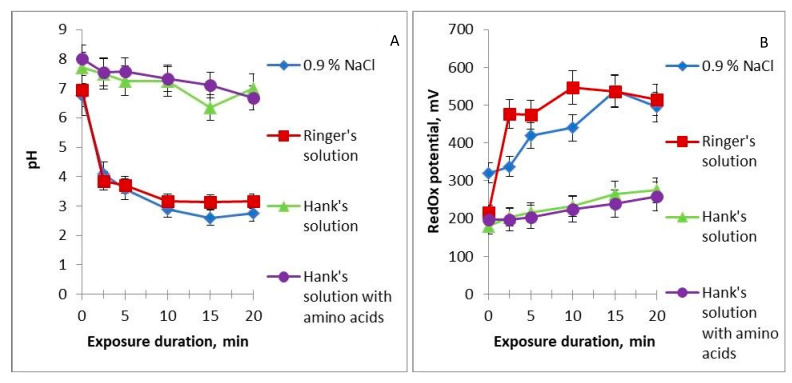
Dependence of pH (**A**) and redox potential (**B**) of the studied solutions on the time of their treatment with cold plasma.

Before cold plasma treatment, all the studied solutions have a neutral pH. Already at 2.5 min of treatment, the pH of the NaCl solution and Ringer’s solution drops to 4 units and continues gradually decreasing (Figure 3A). The pH of HBSS and HBSS with amino acids decreases ever so slightly after 20 min of treatment. These results allow dividing the studied solutions into two groups, one having strong buffering properties and keeping the pH neutral and the other having weak buffering properties and acquiring acidic properties at a short time of treatment with cold plasma. After adding the PTS to the nutrient medium with cells, its pH remained neutral.

The redox potential increases linearly in all solutions with increasing time of their treatment with cold plasma, but at different rates (Figure 3B). Redox potential measures the ability of components of a particular solution to recover. The higher the redox potential, the more atoms with variable valence in the highest oxidation state are contained in the solution. When subjected to cold plasma, nitrogen atoms are oxidised, forming nitrogen oxides, nitrite ions, and nitrate ions, and we assume that these processes are the main factors in the increase in the redox potential in our case. In the Ringer’s solution containing K^+^, Ca^2+^, Na^+^, and Cl^−^ ions, redox potential increases the fastest, with saturation observed after 10 min of treatment; in 0.9% NaCl solution, oxidation is slightly slower, reaching the same value as in Ringer’s solution after 15 min. The flattening of the curve at long treatment times may be associated with limitations on the number of oxidisable atoms in the solution. Redox potential increases to a much lesser extent in HBSS and HBSS with amino acids. This difference again correlates with the buffer properties of the solutions.

### 2.2. Cell Vitality When Subjected to PTS Alone and in Combination with Medications

Doxorubicin, medroxyprogesterone acetate (MPA), and all solutions treated with cold plasma showed an effect on cell vitality proportional to the treatment time; this was expressed in apoptosis in one case and increased lysosomal activity in the other.

**Figure 4:**
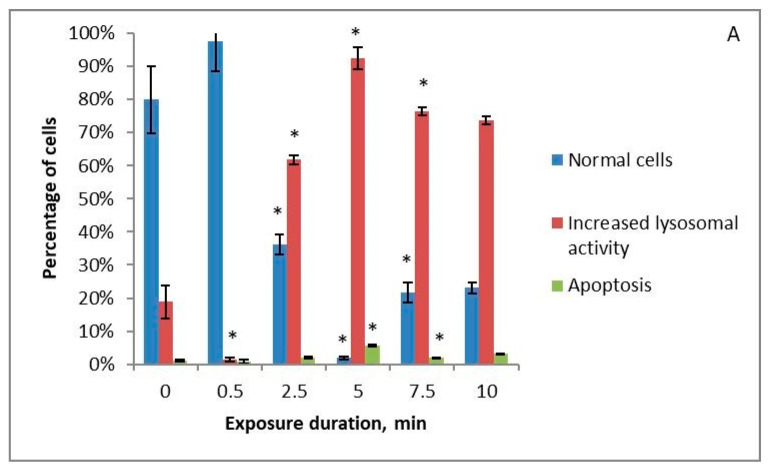

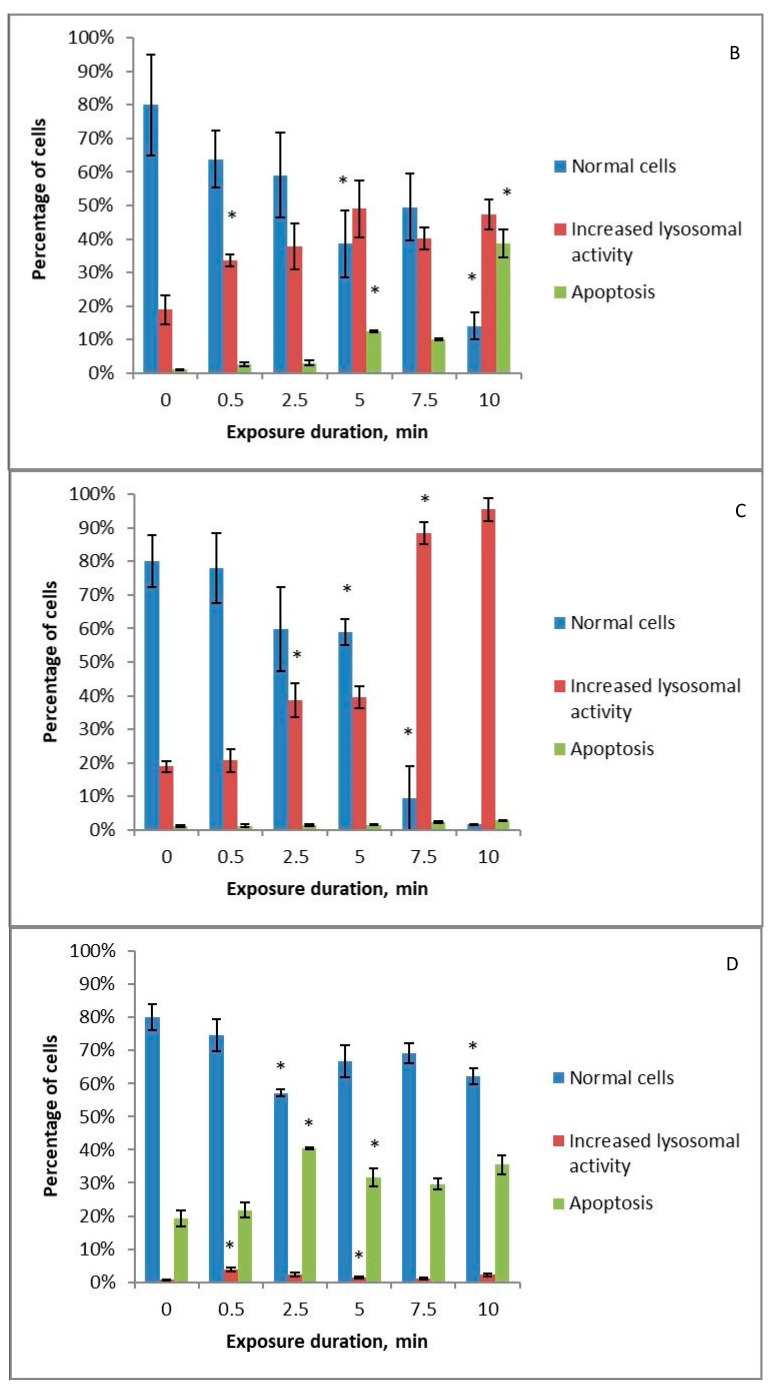
Changes in the apoptotic and increased lysosomal activity of K562 myeloid leukaemia cells under the influence of 0.9% NaCl solution (**A**), Ringer’s solution (**B**), and Hank’s Balanced Salt Solution (**C**) or Hank’s Balanced Salt Solution with the addition of amino acids in physiological concentrations (**D**) treated with piezoelectric direct discharge for a specific period of time without pharmaceuticals to the nutrient medium. The * sign indicates a statistically significant difference from a shorter treatment time (*p* < 0.05).

All the studied solutions that were treated with cold plasma stimulate an increase in lysosomal activity in cells with increasing treatment time. When using Ringer’s solution (Figure 4B), long treatment times result in increased lysosomal activity being replaced by apoptosis. HBSS (Figure 4C) and 0.9% NaCl solution (Figure 4A) treated with cold plasma stimulate only the increase in lysosomal activity. The level of apoptotic cells at all treatment times remains low for these three solutions. HBSS with amino acids (Figure 4D) treated with cold plasma for 2.5 min or more lowers cell vitality by an average of 20%, while the level of lysosomal activity remains low at all treatment times.

The development of apoptosis under long Ringer’s solution treatment times can be associated with its earliest studied solutions reaching the maximum redox potential. Despite the drop in pH, the 0.9% NaCl solution did not shift towards apoptosis under long treatment times. In addition, the nutrient medium to which solutions treated with cold plasma are added has a sufficiently powerful buffer system to equalise the pH. One of the reasons for the increase in the apoptotic response may therefore be the accumulation of nitrates in Ringer’s solution.

We have previously evaluated the autophagy level (expressed in the level of lysosomal activity) and apoptosis level for 0.9% NaCl solution treated with piezoelectric direct discharge in K562, Jurkat cell lines, and healthy mononuclear leukocytes [39]. In that experiment, we observed an increase in lysosome activity at medium doses and the development of apoptosis at high treatment times for all three types of cells.

**Figure 5:**
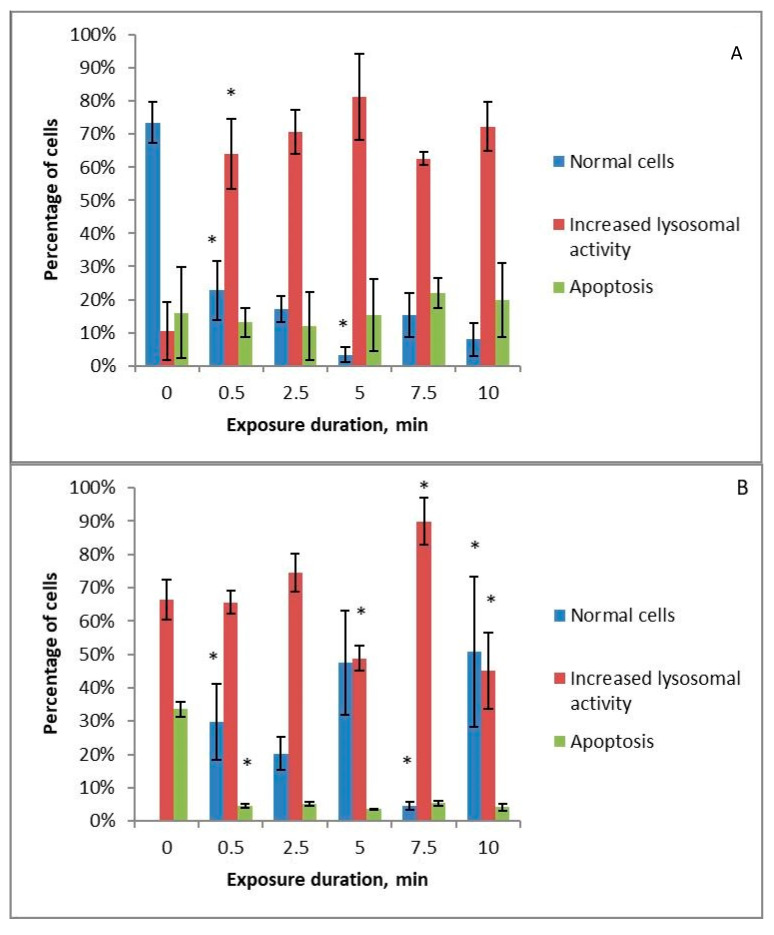

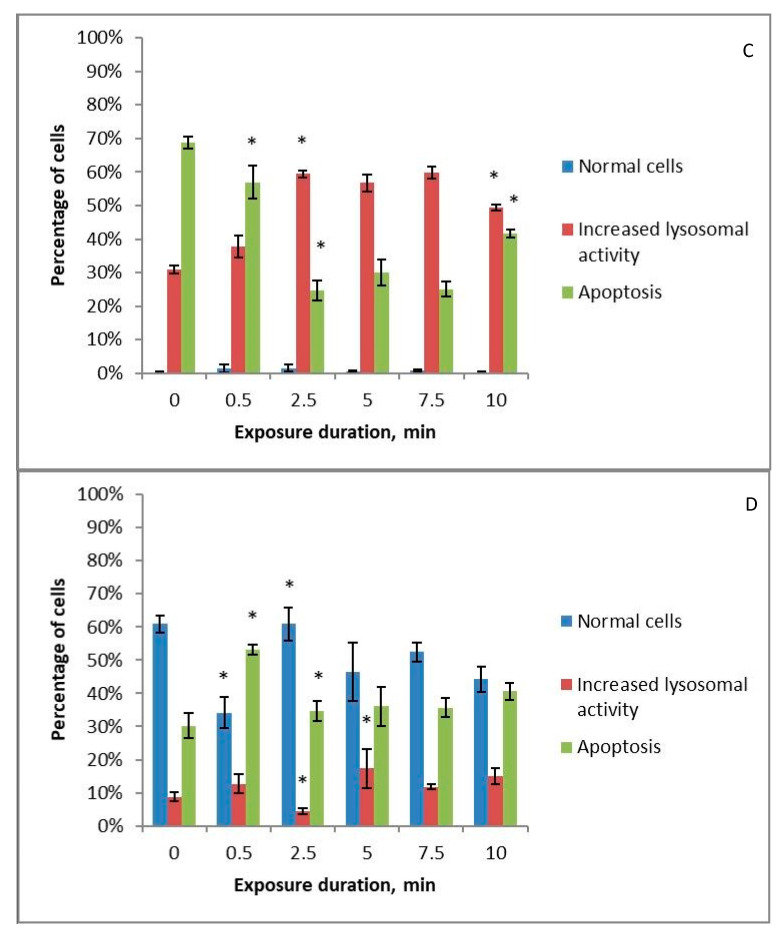
Changes in the apoptotic and increased lysosomal activity of K562 myeloid leukaemia cells under the influence of 0.9% NaCl solution (**A**), Ringer’s solution (**B**), and Hank’s Balanced Salt Solution (**C**) or Hank’s Balanced Salt Solution with the addition of amino acids in physiological concentrations (**D**) treated with piezoelectric direct discharge for a specific period of time with the simultaneous addition of doxorubicin to the nutrient medium. The * sign indicates a statistically significant difference from a shorter treatment time (*p* < 0.05).

Doxorubicin has a marked cytotoxic effect on cells, inducing apoptosis in them (Figure 5A–D). However, the addition of cold-plasma-treated solutions to the nutrient medium neutralises the effect of doxorubicin, that is presumably related to the direction of cells along the autophagy pathway. Cell survival rate increases with increasing treatment time, as does the activity of lysosomes in them. The results differ only for HBSS with amino acids (Figure 5D): the apoptosis level for the treated solution is statistically higher than without cold plasma treatment at 0.5 and 10 min.

The absence of the dependence of cell survival on the solution treatment times is at odds with the results obtained in numerous studies [16,40,41]. This difference may be since small differences in the physicochemical characteristics of PTS lead to completely different biological effects. The main biologically active components of PTS are considered to be NO_2_^−^, NO_3_^−^, NO, H_2_O_2_, and ONOO^−^. However, in this work, serious differences in the concentration of nitrites and hydrogen peroxide, as well as in pH and redox potential, did not lead to such differences in the biological effect for NaCl solution, Ringer’s solution, and HBSS. It was previously shown that a solution treated with cold plasma to form hydrogen peroxide, nitrites, and nitrates has a lower cytotoxic effect than a chemically prepared solution of hydrogen peroxide, nitrites, and nitrates at the same concentrations [42,43]. It is possible that any particular biological effects are caused not by specific reactive oxygen and nitrogen species (RONS) but by their specific concentration ratios and molecular energies. In our work, these seemingly insignificant factors potentially caused the predominance of one of the mechanisms: in one case, a cascade triggering apoptosis, and in the other, a cascade causing the destruction of damaged organelles by the cell, autophagy. In the second case, the cell has a chance to clean up and survive if the damage to the organelles is relatively limited. An important research area in modern oncology is the inhibition of the autophagy mechanism and the initiation of apoptosis [44,45]. Some studies on acute myeloid leukaemia, however, suggest that cancer cell differentiation is stimulated through the initiation of autophagy [46].

**Figure 6:**
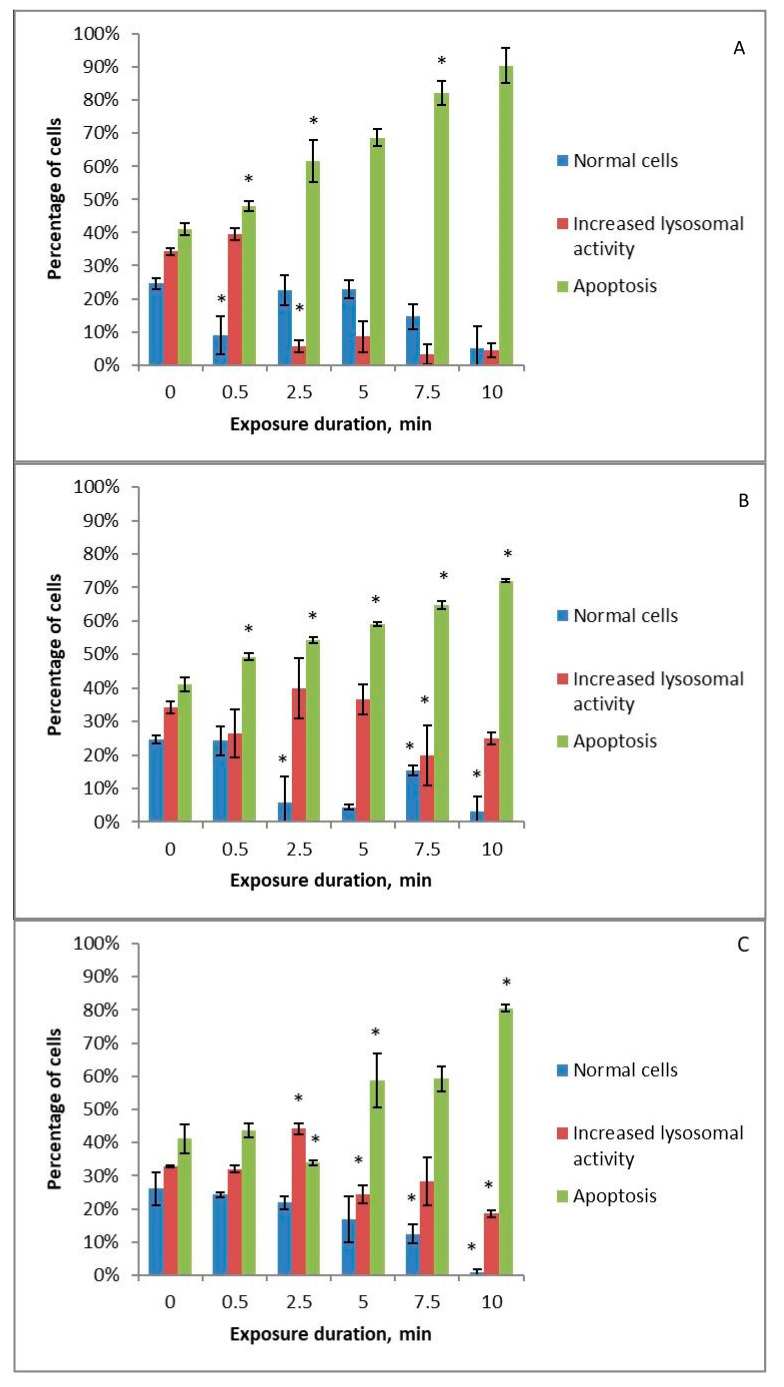

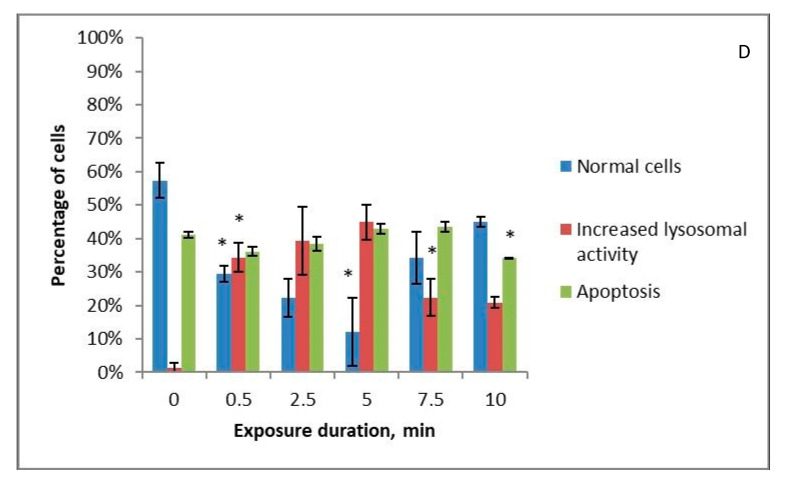
Changes in the apoptotic and increased lysosomal activity of K562 myeloid leukaemia cells under the influence of 0.9% NaCl solution (**A**), Ringer’s solution (**B**), and Hank’s Balanced Salt Solution (**C**) or Hank’s Balanced Salt Solution with the addition of amino acids in physiological concentrations (**D**) treated with piezoelectric direct discharge for a specific period of time with simultaneous addition of medroxyprogesterone acetate to the nutrient medium. The * sign indicates a statistically significant difference from a shorter treatment time (*p* < 0.05).

MPA gave a completely different result: the addition of solutions treated with cold plasma to the nutrient medium enhanced the apoptotic effect of MPA in all cases (Figure 6A–C), except for HBSS with amino acids (Figure 6D), where treatment with cold plasma does not reduce cell vitality and at medium treatment time only enhances increased lysosomal activity.

Tomić et al. studied the stimulation of autophagy through PTS [7]. However, they argued that this is not the predominant mechanism. Yoshikawa et al. point to the important role of autophagy in the anti-cancer effect of PTS [47]. Zhen et al. reported autophagy of damaged mitochondria in pancreatic cancer cells after PTS treatment [48]. Zahedian et al. demonstrated that PTS in combination with doxorubicin could induce apoptotic or necrotic cell death depending on the cold plasma treatment time, whereas, alone, PTS increased apoptosis in proportion to the treatment time [49].

The main biological effects of doxorubicin are the formation of reactive oxygen species inside the cell and DNA damage [32]. Doxorubicin can also indirectly stimulate the conversion of sphingomyelin into ceramide, which positively affects the initiation of apoptosis [32]. Doxorubicin is also able to activate autophagy because it inhibits the action of the mammalian target of rapamycin (mTOR), which regulates the initiation of autophagy, and activates Beclin-1, which is responsible for the formation of autophagosomes [31]. MPA inhibits the activity of the transforming factor β and oestrogens on cancer cells, weakens Ca^2+^- and cGMP-mediated proliferative signals to the nucleus, inhibits the activity of multidrug resistance proteins, induces apoptosis and necrosis through the formation of intracellular RONS and the C/EBP homologous protein transcription factor (CHOP) and the forkhead box protein O1 (FOXO1) mechanisms, and, when combined with doxorubicin, blocks the G2/M-phase of the cell cycle [36]. Mitochondrial and DNA damage can inhibit mTOR activity and stimulate autophagy. The accumulation of adenosine monophosphate (AMP) in the cell results in the AMP protein kinase inhibiting mTOR, thereby inducing autophagy [50].

**Figure 7:**
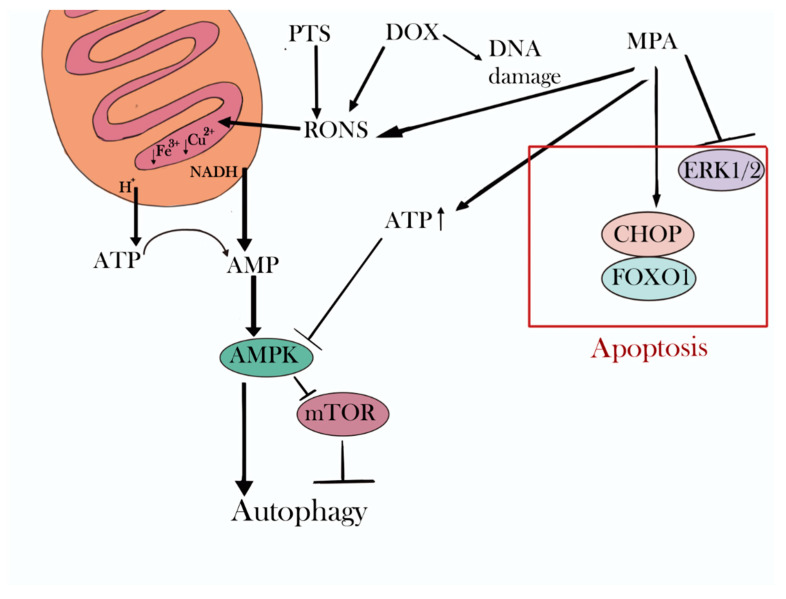
Breakdown of hypothetical processes in the cell caused by medroxyprogesterone acetate (MPA), doxorubicin (DOX), and a cold-plasma-treated solution (PTS). ERK1/2—the extracellular signal-regulated kinase pathways; CHOP—the C/EBP homologous protein transcription factor; FOXO1—the forkhead box protein O1; RONS—reactive oxygen and nitrogen species; mTOR—the mammalian target of rapamycin; AMPK—adenosine monophosphate activated protein kinase.

Our hypothesis about the mechanisms of the joint action of PTS and doxorubicin is as follows (Figure 7). The main cytotoxic effect of doxorubicin is associated with DNA intercalation and, to a lesser extent, provided by RONS generation in the cell. PTS, on the other hand, operates mainly through RONS. We can, therefore, assume that it is the RONS in the concentrations that we obtained that stimulate the development of autophagy. Mitochondria are the main target for RONS. By penetrating them, RONS can, on the one hand, reduce the membrane potential due to binding to protons and, on the other hand, restore NADH and variable-valence metals in the respiratory chain complexes. Such processes will reduce ATP synthesis and cause the accumulation of AMP, which will stimulate autophagy development. As mentioned above, doxorubicin also causes autophagy, but to a very limited extent, which may be due to the low production of RONS and a high degree of cell protection from them. RONS are normally constantly generated in cells, giving the cell a large number of RONS-neutralising protective antioxidant mechanisms. In this regard, RONS contained in PTS and generated by doxorubicin do not cause severe toxic effects. K562 myeloid leukaemia cells have specific receptors for progestins [51] through which certain signalling cascades are triggered in the cell. As such, the ligand binding to the mitochondrial progesterone receptor induces increased ATP production, which can neutralise the effect of RONS [52].

The predominance of apoptosis when using HBSS with the addition of amino acids as a PTS can be explained by the protective properties of amino acids that interact with radicals and change their chemical structure [53]. As a result, the RONS appearing in the nutrient medium interact not with cellular structures but with amino acids and are neutralised.

## 3. Materials and Methods

### 3.1. Materials

In this experiment, we used 0.9% NaCl saline solution (LLC NPP PanEco, Cat. No. R011p, Moscow, Russia), Ringer’s solution (LLC Gematek, No. LS-001550, Tver’, Russia), Hank’s Balanced Salt Solution (LLC NPP PanEco, Cat. No. R020p, Moscow, Russia), RPMI nutrient medium (LLC NPP PanEco, Cat. No. C350, Moscow, Russia), foetal bovine serum (LLC NPP PanEco, Cat. No. FB-1001, Moscow, Russia), xylenol orange (JSC Lenreaktiv, 100544, Sankt-Peterburg, Russia), sulfuric acid (LLC DO KhRS, 7664-93-9, Ufa, Russia), Mohr’s salt (LLC ORT Khimreaktivy, 4208-72, Ekaterinburg, Russia), sorbitol (PanReac-AppliChem, A-2222, 0500f, Chicago, IL USA), Griess reagent (JSC Lenreaktiv, 373131L, Sankt-Peterburg, Russia), doxorubicin (LLC TH Chimmed, D063-100MG, Moscow, Russia), medroxyprogesterone acetate (LLC TH Chimmed, Cat. No. MG00-100, Moscow, Russia), acridine orange (LLC NPP PanEco, Cat. No. 0050, Moscow, Russia), MTT (thiazolyl blue tetrazolium bromide) (PanReac-AppliChem, A-2231, 0010, Chicago, IL USA), amphotericin (LLC NPP PanEco, Cat. No. A006, Moscow, Russia), and dimethyl sulfoxide (DMSO) (Tatkhimfarmpreparaty, No. LSR-003126/08, Kazan’, Russia).

### 3.2. Cold Plasma Treatment of Solutions

In this work, cold plasma was used to treat solutions with a CAPKO (Cold Atmospheric plasma of Kolik) source developed at the GPI RAS (General Physics Institute of the Russian Academy of Sciences) [54]. The characteristics of the source are given in more detail in [55]. The 5 mL treated solution was placed in the wells of a 6-well tablet with a diameter of 6 cm. The piezotransformer was brought to a distance of 5 mm from the surface of the solution, after which a piezo discharge was generated between the piezotransformer and the surface of the solution. The treatment was carried out at a temperature of 25 °C and a humidity level of 40%. The treated solution was also plasma-heated to 40 ± 3 °C. The processed solutions are 0.9% NaCl, Ringer’s solution, HBSS, and HBSS with amino acids at concentrations compatible with blood (Table 1).

### 3.3. Determination of Hydrogen Peroxide Concentration

To determine the concentration of hydrogen peroxide, the test solution was diluted with an untreated solution; FOX reagent (250 mM H_2_SO_4_, 1 mM xylenol orange, 1 mM Mohr’s salt, 0.5 M sorbitol) was added in a 1:1 volume ratio [57]. Then, 10 min after FOX was added, the optical density of the solution was measured at a wavelength of 562 nm, which is proportional to the concentration of hydrogen peroxide.

The data obtained were confirmed by measuring the optical density of the solution at 200 nm [58]. A spectrophotometer Cintra 4040 with cuvettes was used for all concentration measurements.

### 3.4. Determination of the Nitrite Ion Concentration

A 5% solution of the Griess reagent in 12% acetic acid was added to the test solution. The optical density of the solution was measured at a wavelength of 530 nm, which is proportional to the concentration of nitrite ions [59].

### 3.5. Determination of the Hypochlorite Concentration

The optical density of the calibration and test solutions was measured at a wavelength of 292 nm, which is directly proportional to the hypochlorite concentration [60].

### 3.6. Determination of the pH and Redox Potential

The acidity and redox potential of plasma-treated solutions were measured using a pH electrode (HI1131) and a redox electrode (HI3148B) connected to the device “HANNA instruments HI2550”. All measurements were carried out immediately after treatment of the solutions with cold plasma and an hour later. The concentrations of hydrogen peroxide, nitrite, hypochlorite, pH, and redox potential did not change within an hour after treatment.

### 3.7. Cell Culture

The K562 myeloid leukaemia cell culture (obtained from a biocollection of human cell strains of the Federal State Budget Scientific Institution VILAR) was planted in 96-well plates and cultured for a day in the RPMI nutrient medium supplemented with 10% foetal bovine serum. A day later, 0.9% NaCl solution, Ringer’s solution, HBSS, and HBSS with amino acids were treated with a piezoelectric direct discharge and added to the cell-containing medium an hour after treatment to make their final volume equal to 20% of the total of the medium. The final concentration of doxorubicin added to the medium was 5 × 10^−6^ M [61] and that of MPA was 10^−5^ M [62] (a cytotoxic effect was shown). The studied cells were incubated in a thermostat with an increased concentration of CO_2_ (5%) at a temperature of 37 °C. The autophagy and apoptosis levels were assessed 48 h after adding the active substances.

### 3.8. Assessment of Lysosomal Activity and Apoptosis Levels

The overall vitality of the cells was assessed using the MTT (the 3-(4, 5-dimethylthiazol-2-yl)2, 5-diphenyl-2H-tetrazolium bromide assay) method based on the reduction of tetrazolium dye by NADP-dependent and glycolytic leukocyte enzymes [63]. To assess the lysosome activity level, microslides stained with acridine orange were made [64]. These were photographed with a fluorescent microscope, after which the photographs were evaluated using the ImageJ program. Acridine orange bound to DNA and RNA fluoresces green, and inside acidic active lysosomes, yellow and orange. By calculating the percentage ratio of the number of orange and yellow pixels in the photo to the number of green pixels, it is possible to estimate the lysosome activity level in the cells. We believe that this lysosome activity level corresponds to the autophagy level in the sample. It was also possible to observe the fragmentation of cell nuclei in the apoptotic cells and the formation of apoptosomes, which corresponds to one or more mechanisms of cell death [64,65].

### 3.9. Statistical Data Processing

Each experiment was repeated 3 times, with 6 repetitions within each experiment. The mean ± standard deviation value was calculated for each point. A statistically significant difference (*p* ≤ 0.05) was calculated using the Mann–Whitney criterion and is shown in the figures with *.

## 4. Conclusions

In this paper, the first-ever investigation of the cytotoxic effect of plasma-treated solutions in combination with anti-cancer drugs on myeloid leukaemia cells was carried out. Four isotonic solutions similar in composition to blood plasma were chosen as the basis for PTS synthesis, while doxorubicin and medroxyprogesterone acetate were used as anti-cancer drugs. It was shown through various analyses that the effects of the combined action of various solutions treated with cold plasma and a particular medicinal substance can differ greatly from each other. In combination with doxorubicin, PTS weakens its cytotoxic effect; then, in combination with MPA, it enhances the effect. A model was developed to explain the results of the experiments. In light of the available results, there is a need to carry out further in vivo studies of intra-cell molecular processes to develop a more comprehensive understanding of the combined action of PTS and anti-cancer drugs.

## Figures and Tables

**Table 1:** Concentrations of amino acids added to Hank’s Balanced Salt Solution [56].

Amino Acid	Concentration, μM
Tyrosine	70
Tryptophan	50
Methionine	20
Cysteine	50
Histidine	90
Proline	250
Phenylalanine	70

## Data Availability

The data presented in this study are available on request from the corresponding author. The data are not publicly available due to privacy.
